# Distribution of Hepatitis C Virus Genotypes in Iranian Chronic Infected Patients

**DOI:** 10.5812/hepatmon.7991

**Published:** 2013-01-20

**Authors:** Fatemeh Jahanbakhsh Sefidi, Hossein Keyvani, Seyed Hamidreza Monavari, Seyed Moayed Alavian, Shahin Fakhim, Farah Bokharaei-Salim

**Affiliations:** 1 Department of Virology, Tehran University of Medical Sciences, Tehran, IR Iran; 2 Baqiyatallah Research Center for Gastroenterology and Liver Diseases, Tehran, IR Iran; 3 Department of Civil Engineering, Islamic Azad University, Shahre Qods, Tehran, IR Iran

**Keywords:** Hepatitis C, Genotype, Iran

## Abstract

**Background:**

Hepatitis C virus (HCV) has different genotypes throughout the world. Since the determination of which antiviral treatment to be applied is related to HCV genotypes, identification of an individual’s HCV genotypes prior to antiviral therapy is critical.

**Objectives:**

The purpose of this study was to investigate the distribution of HCV genotypes in a large population of Iranian HCV infected patients.

**Patients and Methods:**

Eleven thousand, five hundred and sixty one patients with chronic HCV infection which referred to hospitals related to the Tehran University of Medical Sciences and Tehran Hepatitis Center-Clinical Department of Baqiyatallah Research Center for Gastroeneterology and Liver Disease from March 2003 to December 2011 were enrolled. Following extraction of viral RNA of the serum, HCV-RNA was detected using reverse transcriptase-nested polymerase chain reaction (RT-nested PCR) and then HCV genotypes analyzed by restriction fragment length polymorphism (RFLP) assay.

**Results:**

The mean age of patients was 37.6 ± 14.2 years (range: 1-87). The highest frequency was noted for subtype 1a (44.9%) followed by subtype 3a (39.6%), and 1b (11.3%). Mixed HCV genotypes were also found in 2.5% of the total cases. Subtype 1a was the most frequent genotype in patients over 40 years of age (46.1% versus 42.4%) and subtype 3a was the most frequent in patients under 40 years old (41.5% versus 38.9%).

**Conclusions:**

This study suggested that the dominant HCV subtype among Iranian patients was 1a followed by subtype 3a.

## 1. Background

Hepatitis C Virus (HCV) is an enveloped RNA virus with positive polarity belonging to the family Flaviviridae and genus hepacivirus (1). Infection with HCV is the most common cause of chronic liver disease and hepatocellular carcinoma. This infection is a virulent health problem affecting more than 170 million people worldwide. The prevalence rate of HCV infection is from 0.2 up to 40% in different countries (2, 3) (about 0.2 to 2.2% in developed countries and nearly 7% in developing countries) (4). The prevalence of HCV infection is less than 0.5% (0.1% in women and 1.0% in men) in the general population of Iran (5) and this infection is common in hemophilia, thalasemia, hemodialysis patients and in intravenous drug users (IVDU) (6, 7).There are six large distinct genotypes (termed 1 to 6), in addition to over 70 different subtypes (termed a, b, c, etc.) which are distributed worldwide. It has been established that 30% to 33% of the genotypes differ from each other, as do 20 to 25% of the subtypes (8). According to the available information in Iran, the most predominant HCV subtype is 1a followed by 3a and 1b (9-12). HCV genotyping is a fundamental tool in both the epidemiology and the management of HCV infected patients and assists with understanding the evolution of the virus and epidemiology of the disease in relation to demography, risk profile and severity of the disease process. Different genotypes seem to be associated with diverse severity magnitudes of the liver damage and responses to antiviral treatment (13). In recent years, a shift in the prevalence of HCV genotypes has been reported in a number of countries around the world (14-16). The shift in the genotype circulation may have a significant impact on clinical management of the patients as well as on the public health. The purpose of this study was to investigate the distribution of HCV genotypes in a large population of Iranian HCV infected patients.

## 2. Objectives

The main objective of the current study was to investigate the distribution of HCV genotypes among a large population of chronically HCV infected patients prior to antiviral therapy.

## 3. Patients and Methods

### 3.1. Study Population

From March 2003 to December 2011, a total of 11561 patients who were anti-HCV antibody and HCV-RNA positive selected for antiviral treatment were enrolled in this cross sectional study. They had been referred to the Hospitals related to Tehran University of Medical Sciences and Tehran Hepatitis Center-Clinical Department of Baqiyatallah Research Center for Gastroeneterology and Liver Disease. The study was approved by the local ethics committee of Gastrointestinal and Liver Disease Research Center (GILDRC) of Tehran University of Medical Sciences. Informed consent was obtained from each patient. Inclusion criteria were positive anti-HCV antibodies along with positive plasma HCV RNA. Moreover, none of thepatients under study had been previously treated for hepatitis C virus infection.

### 3.2. Samples Collection

About 5 mL of peripheral blood was collected from each patient into sterile EDTA-containing vacutainer tubes and then plasma was separated and stored at −70°C for later detection.

### 3.3. RNA Extraction and Genotyping

To determine the genotype of HCV, viral RNA was extracted from 140 μl of plasma using the QIAamp Viral RNA Extraction kit (Qiagen GmbH, Hilden, Germany) according to the manufacturer’s instructions. Detection of HCV-RNA in plasma was carried out using reverse transcription-nested polymerase chain reaction (RT-nested PCR) (17). Briefly, cDNA synthesis of HCV-RNA was performed as follows: 5 μl of extracted RNA was added to a reagent mixture which contained 20 pmol of random hexamer, 200 U of Moloney Murine Leukemia Virus Reverse Transcriptase (Fermentas GmbH, Germany), 8 units of RNase inhibitor (Fermentas GmbH, Germany), 125 µmol mix deoxynucleotide triphosphat (Fermentas GmbH, Germany), 1 μL of diethyl-pyrocarbonate (DEPC) treated water, as well as 4 μl of 5X reverse transcriptase reaction buffer. The volume was adjusted to 20 μl. The reactant was incubated at 42°C for 30 minutes and then followed by heating at 72°C for 10 minutes in order to inactive reverse transcriptase. Hepatitis C virus genotypes were determined in RT-nested PCR products using restriction fragment length polymorphism (RFLP) assay of the 5´-UTR of the HCV genome as described earlier (17). PCR products (173-bp) of specimens with digested PCR products by restriction enzymes, positive and negative controls and DNA size marker (50bp) were visualized by 2.5% agarose gel electrophoresis. HCV Genotypes were determined based on the molecular weight of each product. 

### 3.4 Statistical Analysis

Data were analyzed using SPSS version 16 and descriptive statistical methods such as cross tabulation, t-student and chi-square were used.

## 4. Results

Eleven thousand, five hundred and sixty one patients with established chronic HCV infection were recruited in this cross sectional study. The genotype of these patients was detected prior initiating the treatment. Out of 11561 patients, 9645 (83.4%) were male. Demographic and HCV genotype distribution of all patients over the study periods are presented in Table 1. The mean age of patients was 37.6 ± 14.2 years (range: 1-87). The mean age of the women was statistically higher than men (38.5 versus 36.6, respectively) (T-test, P < 0.05). The majority of the patients aged 16-30 years, followed by 31-45 years. The average age at the time of treatment revealed a decreasing trend since 2003 (from 37.3 ± 14.6 in 2003 to 40.3 ± 13.2 in 2011). The most common genotype in the study population was subtype 1a (44.9%) followed by subtype 3a (39.6%), and 1b (11.3 %). Mixed HCV genotype infection was observed in 2.5% of the study population. Of 292 mixed genotype infection cases, mixed inter-genotype infection 1a and 3a was the most common (56.5%). On the other hand, the mixed infection with three HCV genotypes was detected in 1.7% of these patients, as presented in Table 2. Multiple HCV genotypes were found in 189 (64.7%) patients with multiple blood transfusions. Subtype 1a was the most frequent genotype in patients over 40 years of age (46.1% versus 42.4%) and subtype 3a was the most frequent in patients under 40 years old (41.5% versus 38.9%). Subtype 1b was more frequent (1b: 13.6%) in female patients whereas subtype 3a was more frequent (3a: 42.8.1%) in male cases. It should also be noted that the difference in the distribution of the genotypes between males and females was statistically significant (P = 0.001). The frequencies of the subtypes 1a and 1b were higher in participants over 40 years of age (1a: 46.1% versus 42.4%, 1b: 12.7% versus 10.7%). In the age group under 40, subtype 3a was common (41.5% versus 38.9%).

**Table 1:** Demographic Parameters and Hepatitis C Virus Genotypes Distribution of Patients with Hepatitis C Virus Infection From March 2003 to December 2011

Year	2003	2004	2005	2006	2007	2008	2009	2010	2011	Total
**Total No.**	777	977	1016	1142	1872	1557	1515	1365	1339	11561
**Male/Female, No.**	626/151	817/160	851/165	942/200	1575/297	1315/241	1282/233	1135/230	1102/238	9645/1915
**Age, y, Mean **±** SD**	37.3 ± 14.6	38.4 ± 15.2	39.8 ± 14.2	39.6 ± 13.5	35.3 ± 14.0	36.0 ± 15.4	37.3 ± 13.5	38.9 ± 13.5	40.3 ± 13.2	37.9 ± 14.2
**Type of Subtypes and Genotypes, %**										
Subtype 1a	47.8	49.9	42.4	44.6	46.2	44.7	45.4	44.0	40.2	44.9
Subtype 3a	30.1	34.9	39.7	39.3	39.0	43.1	41.0	42.3	41.6	39.6
Subtype 1b	18.3	13.0	10.2	10.0	9.1	9.1	9.9	11.1	14.9	11.3
Genotype 2	0.8	0.6	1.0	1.3	0.6	0.4	0.6	0.5	0.9	0.7
Subtype 3b	0.0	0.1	0.0	0.9	0.7	0.2	0.0	0.0	0.0	0.2
Genotype 4	0.4	0.1	0.6	0.6	0.8	1.1	0.7	1.2	0.9	0.8
Mixed infection	2.6	1.4	6.1	3.3	3.6	1.4	2.4	0.8	1.6	2.5

**Table 2:** Hepatitis C Virus Genotypes Distribution of 292 Patients with Hepatitis C Mixed Infection

HCV mixed infection	No. (%)
**Subtypes 1a, 1b**	46 (15.8)
**Subtypes 1a, 1b, 3a**	4 (1.4)
**Genotype 2, Subtype 1a**	3 (1.0)
**Genotype 2, Subtypes 1a, 3a**	1 (0.3)
**Subtypes 1a, 3a**	165 (56.5)
**Genotype 4, Subtype 1a**	6 (2.1)
**Genotype 2, Subtype 1b**	6 (2.1)
**Subtypes 1b, 3a**	45 (15.4)
**Subtypes 1b, 3b**	1 (0.3)
**Genotype 4, Subtype 1b**	2 (0.7)
**Genotype 2, Subtype 3a**	9 (3.1)
**Subtypes 3a, 3b**	2 (0.7)
**Genotype 4, Subtype 3a **	1 (0.3)
**Genotype 4, Subtype 3b**	1 (0.3)
**Total**	292 (100)

Abbreviations: HCV, hepatitis C virus

## 5. Discussion

The present study was performed on 11,561 chronically HCV infected patients in Iran to evaluate the distribution of HCV genotypes within this population. In the present study, subtype 1a was the most common subtype (44.9%) followed by subtype 3a (39.6%), and subtype 1b (11.3%) (Table 1). Multiple HCV genotypes were detected in serum specimen of 2.5% of the cases and mixed infection by subtypes 1a and 3a was found in 56.5% of these patients (Table 2). The distribution of HCV subtypes in Iranian chronically HCV infected patients were found to be 1a (47%) and 3a (36%) (18) and in a study on a smaller sample size (19) the dominant HCV subtypes were 1a (47%) and 3a (27%). Significantly, the distribution of HCV genotype in the present study was very similar to other Iranian studies (10, 18, 19) but is different from observations in other Middle Eastern countries. As an example the prevalence of genotype 4 in Iran is lower when compared to the majority of Middle East countries including the neighboring Arabic countries (10, 20, 21). The most common genotype in Iraq (22), Saudi Arabia and Kuwait (23), Yeman (24) is genotype 4. The HCV genotype 4 in Iran is prevalent in hemodialysis patients and is related to communication with the neighbor’s countries such as Iraq and Saudi Arabia during the Hajj ceremony (6, 7). The predominant HCV subtype is 1b in Turkey (20, 25), subtype 3a in Pakistan (26, 27) and subtype 1b in Russia (28). It is probable that differences in the race, routes of transmission, and different socioeconomic factors might explain this variation. This study revealed that the frequency of HCV subtypes 1a and 1b was higher in HCV infected patients older than 40 years (1a: 46.1%; 1b: 12.7%) while subtype 3a was most prevalent in patients younger than 40 (41.5 %). Our findings are complemented by several recent studies which indicate an increase in the frequency of HCV 3a in the younger population of Iran (29) Germany (14), Serbia and Montenegro (16), and Slovenia (15). In the current study, the majority of the patients were aged 16-30 years, followed by of 31-45 years which is consistence with the mean age of HCV infection in Iran (30). The mean age of the whole study population at the time of treatment was 37.9 ± 14.2 years. However, the average age of the women was statistically higher than men (38.5 versus 36.6, respectively) (T-test, P<0.05) which could indicate that the main risk factor was transfusion in women and intravenous drug abuse history in men (30). In the last decade, a shift in HCV genotype distribution has been reported in many countries, as an example an increase was seen in the prevalence of the subtypes 3a, 1a, and genotype 4, and a decrease in subtypes 1b and genotype 2 (14, 29, 31). Interestingly, in the current study an increase was detected in the prevalence of the subtypes 3a and genotype 4, and a decrease in subtype 1a and 1b. It should be noted that the route of HCV transmission probably causes this epidemiological change in genotype distribution. Numerous investigators have reported that patients with a history of blood transfusion were mostly infected with subtype 1b while intravenous drug abusers were infected with subtype 3a (13, 30, 32-34). The present study revealed that 1a was the most frequent subtype in older patients (over 40) (46.1%), while the subtype 3a was characterized in younger patients (41.5%). It can be concluded that genotype 1(a/b) is the most dominant HCV genotype in older patients with chronic HCV infection in Iran. The high prevalence of subtype 3a in young patients suggests the increased number of IVDU as the main route of HCV transmission (35). This study led to the finding that HCV is most prevalent in young patients and in men. This can be due to the route of transmission since the main risk factor for transmission of HCV infection is now IVDU, involving shared unsterilized or poorly sterilized needles and syringes (36, 37). Therefore, young male individuals are more susceptible to acquire HCV infection. Mixed HCV infection is infection of an individual with more than one HCV genotypes (35). There are numerous reports indicating that infection with distinct HCV genotype doesn’t make a barrier to infect with other HCV genotypes, thus several exposures to HCV may be lead to multiple episodes and mixed infection in some patients. This infection may result in severe disease, unresponsiveness to antiviral therapy or relapse following the concluding of antiviral treatment (38-41). In the present study the prevalence of mixed HCV infection was about 2.5%, and the relatively high prevalence of mixed inter-genotype HCV infection with 1a and 3a (56.5%) was found in our subjects (Table 2). Mixed HCV infection with two HCV genotypes have been detected in about 1% of HCV positive participants (42, 43), and 1.6 to 31% in patients with multi-transfused hemophiliacs (43, 44). The advantage of this study was its large sample size and long study period which helped to draw a good estimate of HCV genotype distribution in Iran. Furthermore, inclusion of a considerable number of HCV infected children and young adults allowed more precise determination of HCV genotypes distribution according to age. In conclusion, the current study shows that the dominate HCV subtype among Iranian patients is 1a followed by subtype 3a. The results of the present study, on the other hand suggests that the distribution of HCV genotypes is changing, with the increasing of subtype 3a and genotype 4 and a marked decreasing of subtypes 1a and 1b.
